# Effects of early diuretic response to carperitide in acute decompensated heart failure treatment: A single-center retrospective study

**DOI:** 10.1371/journal.pone.0199263

**Published:** 2018-06-18

**Authors:** Yoshitaka Okuhara, Masanori Asakura, Kohei Azuma, Yoshiyuki Orihara, Koichi Nishimura, Tomotaka Ando, Hideyuki Kondo, Yoshiro Naito, Kazunori Kashiwase, Shinichi Hirotani, Masaharu Ishihara, Tohru Masuyama

**Affiliations:** Cardiovascular Division, Department of Internal Medicine, Hyogo College of Medicine, Hyogo, Japan; Osaka University Graduate School of Medicine, JAPAN

## Abstract

**Background:**

Diuretic response is a strong predictor of outcome for admitted patients of acute decompensated heart failure (ADHF). However, little is known about the effects of early diuretic response to carperitide.

**Methods:**

We retrospectively analyzed records of 85 patients hospitalized for ADHF who received carperitide as initial treatment and <40 mg furosemide during the early period. The eligible patients were divided into good diuretic responder (GR) group and poor diuretic responder (PR) group on the basis of median urinary volume.

**Results:**

The PR group demonstrated older age, lower body mass index (BMI), lower estimated glomerular filtration rate, and higher blood urea nitrogen (BUN) level, left ventricular ejection fraction, and β-blockers prescribed at baseline than the GR group. The incidence of worsening renal function (WRF) was significantly higher in the PR group than in the GR group. There was no correlation between early intravenous furosemide dose and urinary volume (Spearman correlation, ρ = 0.111, p = 0.312). Multivariate analysis showed that the statistically significant independent factors associated with poor diuretic response to carperitide were BMI (Odds ratio (OR) = 0.82, 95% confidence interval (CI) 0.68–0.94, p = 0.004) and BUN (OR = 1.07, 95%CI 1.01–1.15, p = 0.018). Kaplan–Meier analysis indicated a lower event-free rate in the PR group than in the GR group (log-rank, *p* = 0.007).

**Conclusions:**

BMI and BUN levels on admission were significant determinants of early poor diuretic response to carperitide. Early poor diuretic response to carperitide was associated with future poor outcomes.

## Introduction

Diuretic response is a useful marker for management of patients hospitalized for acute decompensated heart failure (ADHF). Patients with poor diuretic response have been associated with a higher risk of worse in-hospital outcomes, including worsening renal function (WRF), increase in late mortality, and three-times higher re-hospitalization rates [1]. Poor diuretic response was predicted by lower body mass index (BMI), lower blood pressure (BP), higher blood urea nitrogen (BUN) level, and lower received intravenous diuretic dose [2, 3]. However, almost all patients received intravenous furosemide, and assessment of diuretic response was based on urinary volume or weight loss caused by the intravenous furosemide dose in these registries [3–5].

Carperitide, which is an atrial natriuretic peptide, is recommended in the Japanese Circulation Society guideline for acute heart failure (HF) treatment and is widely used in Japan [6–8]. The acute decompensated HF syndromes (ATTEND) registry, which is the largest HF registry in Japan, revealed that carperitide was used in 69.4% of cases for acute HF treatment during hospitalization [9]. Carperitide induces diuresis and natriuresis by a different mechanism from that by furosemide. However, the diuretic response to carperitide has not been previously reported. The present study aimed to estimate the effects of diuretic response to carperitide.

## Methods

### Patient eligibility

**Fig 1:**
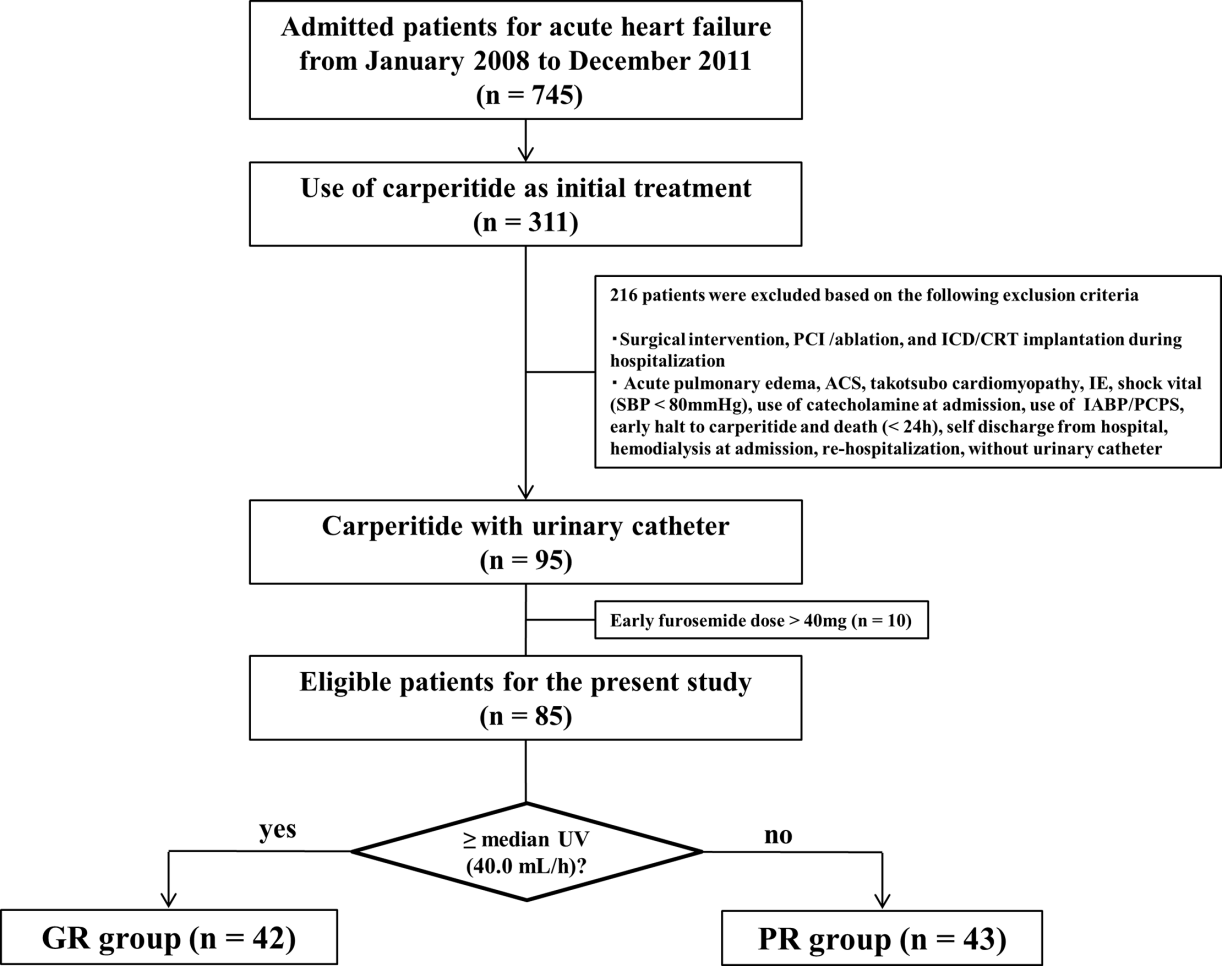
Patient population. PCI, percutaneous coronary intervention; ICD, implantable cardioverter defibrillator; CRT, cardiac resynchronization therapy; ACS, acute coronary syndrome; IE, infectious endocarditis; SBP, systolic blood pressure; IABP, intra-aortic balloon pumping; PCPS, percutaneous cardiopulmonary support; UV, urinary volume; GR, good diuretic responder; PR, poor diuretic responder.

We retrospectively screened 745 hospitalized patients with acute HF who were admitted to the Division of Cardiovascular Medicine at Hyogo College of Medicine between January 2008 and December 2011. Patients were included if they were ≥20 years old, had greater than or equal to New York Heart Association **(**NYHA) class II disease, and received carperitide therapy as an initial treatment. We selected 311 consecutive patients who received intravenous carperitide as an initial treatment. Patients were excluded if they had acute coronary syndrome and takotsubo cardiomyopathy; had systolic blood pressure (SBP) < 80 mmHg; had acute pulmonary edema; required renal replacement therapy (RRT), percutaneous cardiopulmonary support, and intra-aortic balloon pumping at admission; required inotropic agents, including catecholamine and phosphodiesterase III inhibitors, at admission; had an early halt to carperitide therapy and early death within 24 h after admission; checked themselves out of the hospital; underwent surgical intervention, implantable cardioverter defibrillator, and cardiac resynchronization therapy implantation during hospitalization; underwent catheter interventions, including percutaneous coronary intervention and ablation during hospitalization; suffered from infectious endocarditis; were readmitted for ADHF during the study period; or had no urinary catheter from admission because urinary volume measurement is not reliable without a urinary catheter. In addition, we excluded the patients who used total intravenous furosemide > 40 mg from admission until 24:00 of the next day of hospitalization because of the need to minimize the influence of intravenous furosemide. Consequently, a total of 85 patients were eligible for this study (Fig 1).

### Clinical investigations

All data were retrospectively collected from the hospital medical record review. Demographic data, medical histories, laboratory values, echocardiographic findings, and vital signs were collected at admission. Overall treatment strategy, adjustments to carperitide and supplementation of furosemide depending on BP and urinary output were performed at the discretion of the attending physician. All patients were under restricted salt intake (<6 g/day) during hospitalization. The present study complies with the Declaration of Helsinki and was approved by the institutional ethics committee at Hyogo College of Medicine, Hyogo, Japan (approval number 2040). Owing to the retrospective nature of the study, the requirement to obtain informed consent was waived.

### Definitions

The early urinary output was defined as urinary output from admission until 24:00 of the next day of hospitalization because our hospital measures urinary volume per day at 24:00. We regarded the period as the early period in this study. The good diuretic responder (GR) group was defined as greater than or equal to the median urinary output / length of the early period (40mL/h), and the poor diuretic responder (PR) group was defined as less than the median urinary output / length of the early period. Oral loop diuretic doses at admission were converted to furosemide equivalents, with 60 mg azosemide = 8 mg torasemide = 40 mg furosemide. The estimated glomerular filtration rate (eGFR) was calculated by using the Modification of Diet in Renal Disease study equation modified by using a Japanese coefficient: eGFR = 194 × serum creatinine^−1.094^ × age^−0.287^ (×0.739 if female) [10]. WRF was defined as a ≥25% decrease in eGFR from the value at admission until any time during the first 14 days after admission [11–13]. The introduction of RRT because of insufficient diuresis was also included as WRF. Hypotension was defined as SBP < 80 mmHg that required fluid loading, vasopressors, or the reduction or discontinuation of carperitide. The composite outcome was defined as all-cause mortality and re-hospitalization for worsening HF at 1 year after discharge.

### Statistical analysis

All continuous variables are expressed as the mean ± SD if they fit a normal distribution; alternatively, values are expressed as the median and interquartile range (IQR). Categorical variables are expressed as the number (%). Parameters that fit a normal distribution were analyzed by using unpaired t-tests. When the data did not fit a normal distribution, the Mann–Whitney U test was performed. Categorical variables were analyzed by performing the chi-square or Fisher's exact test, as appropriate.

Univariate analyses were used to determine factors predictive of PR and were performed on demographic and clinical factors. Factors from the univariate analyses with *p* ≤ 0.1 were further evaluated in the multivariate logistic model. The relationship between early urinary volume and early furosemide dose was evaluated using the Spearman rank correlation. Kaplan–Meier analysis was used to evaluate the significance of composite outcome differences between the two groups. *P* values were calculated by performing the log-rank test. Results are expressed as odds ratio (ORs) with 95% confidence intervals (95% CI) and *p* values. A *p* value < 0.05 was considered as indicating statistical significance. All statistical analyses were performed by using JMP version 11.2 software (SAS Institute, Cary, NC).

## Results

### Patient population

As shown in Fig 1, the eligible patients were divided into the GR group (n = 42) and PR group (n = 43) that were based on median urinary volume (40.0 mL/h). Baseline characteristics are listed in Table 1. The PR group demonstrated older age, lower BMI, lower eGFR, higher BUN level, higher left ventricular ejection fraction (LVEF), and higher prescription of β-blockers at baseline than the GR group. NYHA class and log NT-pro BNP were not significantly different between the two groups. Rates of the oral loop diuretics and furosemide equivalent doses were similar between the two groups.

**Table 1:** Baseline clinical characteristics.

	GR group	PR group	p value
	(n = 42)	(n = 43)	
Age, median (IQR), y	73 (62–79)	80 (75–86)	<0.001
Male, n (%)	25 (59.5)	25 (58.1)	0.897
Physical examination			
NYHA class III or IV, n (%)	40 (95.2)	41 (95.4)	0.981
Pleural effusion, n (%)	37 (88.1)	37 (86.1)	0.778
De novo heart failure, n (%)	27 (64.3)	24 (55.8)	0.425
BMI, median (IQR), kg/m^2^	23.6 (21.2–27.8)	21.4 (20.4–23.7)	0.007
SBP, mean (SD), mmHg	149 (30)	142 (30)	0.299
Heart rate, mean (SD), beats/min	91 (21)	95 (22)	0.447
Biomarkers			
Hematocrit, mean (SD), %	36.7 (6.0)	36.2 (5.5)	0.711
BUN, median (IQR), mg/dL	21.0 (16.0–24.3)	29.0 (21.0–38.0)	0.002
Serum creatinine, median (IQR), mg/dL	1.05 (0.79–1.34)	1.18 (0.94–1.71)	0.122
eGFR, median (IQR), mL/min/1.73m^2^	49.7 (39.3–59.6)	40.6 (24.6–51.0)	0.039
Serum sodium, median (IQR), mEq/L	140 (136–142)	140 (136–141)	0.961
Serum potassium, median (IQR), mEq/L	4.0 (3.6–4.6)	4.4 (3.9–4.6)	0.145
Serum chloride, median (IQR), mEq/L	104 (100–107)	105 (101–107)	0.520
Log NT-proBNP, mean (SD), pg/mL	3.72 (0.55)	3.81 (0.46)	0.516
Echocardiographic findings			
LVEF, median (IQR), %	34 (27–48)	42 (30–53)	0.049
TRPG, mean (SD), mmHg	38.4 (13.5)	38.7 (12.7)	0.935
E/e prime, median (IQR)	20.5 (15.5–27.9)	26.5 (15.7–41.5)	0.182
Co-morbidities, n (%)			
Hypertension	28 (66.7)	36 (83.7)	0.082
Dyslipidemia	16 (38.1)	14 (32.6)	0.593
Diabetes mellitus	15 (35.7)	18 (41.9)	0.561
Smoking	18 (42.9)	15 (34.9)	0.451
Old myocardial infarction	10 (23.8)	16 (37.2)	0.180
Cardiomyopathy	11 (26.2)	9 (20.9)	0.567
Valvular heart disease	3 (7.1)	4 (9.3)	1.000
Atrial fibrillation	15 (35.7)	18 (41.9)	0.561
Oral medications, n (%)			
Loop diuretics	16 (38.1)	20 (46.5)	0.432
Furosemide equivalent, median (IQR), mg	0 (0–20)	0 (0–20)	0.477
Furosemide equivalent in patients who received furosemide, median (IQR), mg	20 (20–40)	20 (20–40)	0.934
Aldosterone antagonists	10 (23.8)	11 (25.6)	0.850
Thiazide diuretics	2 (4.8)	2 (4.7)	1.000
ACE-inhibitors or ARBs	16 (38.1)	22 (51.2)	0.226
β-blockers	13 (31.0)	24 (55.8)	0.021
Nitrates	8 (19.1)	9 (20.9)	0.828
Digitalis	3 (7.1)	4 (9.3)	1.000

GR, good diuretic responder; PR, poor diuretic responder; IQR, interquartile range; SD, standard deviation; NYHA, New York Heart Association; BMI, body mass index; SBP, systolic blood pressure; BUN, blood urea nitrogen: eGFR, estimated glomerular filtration rate; NT-proBNP, N-terminal pro-B-type natriuretic peptide; LVEF, left ventricular ejection fraction; TRPG, tricuspid regurgitant pressure gradient; ACE-I, angiotensin-converting enzyme inhibitor; ARB, angiotensin receptor blocker.

### In-hospital clinical outcomes and mediations

**Fig 2:**
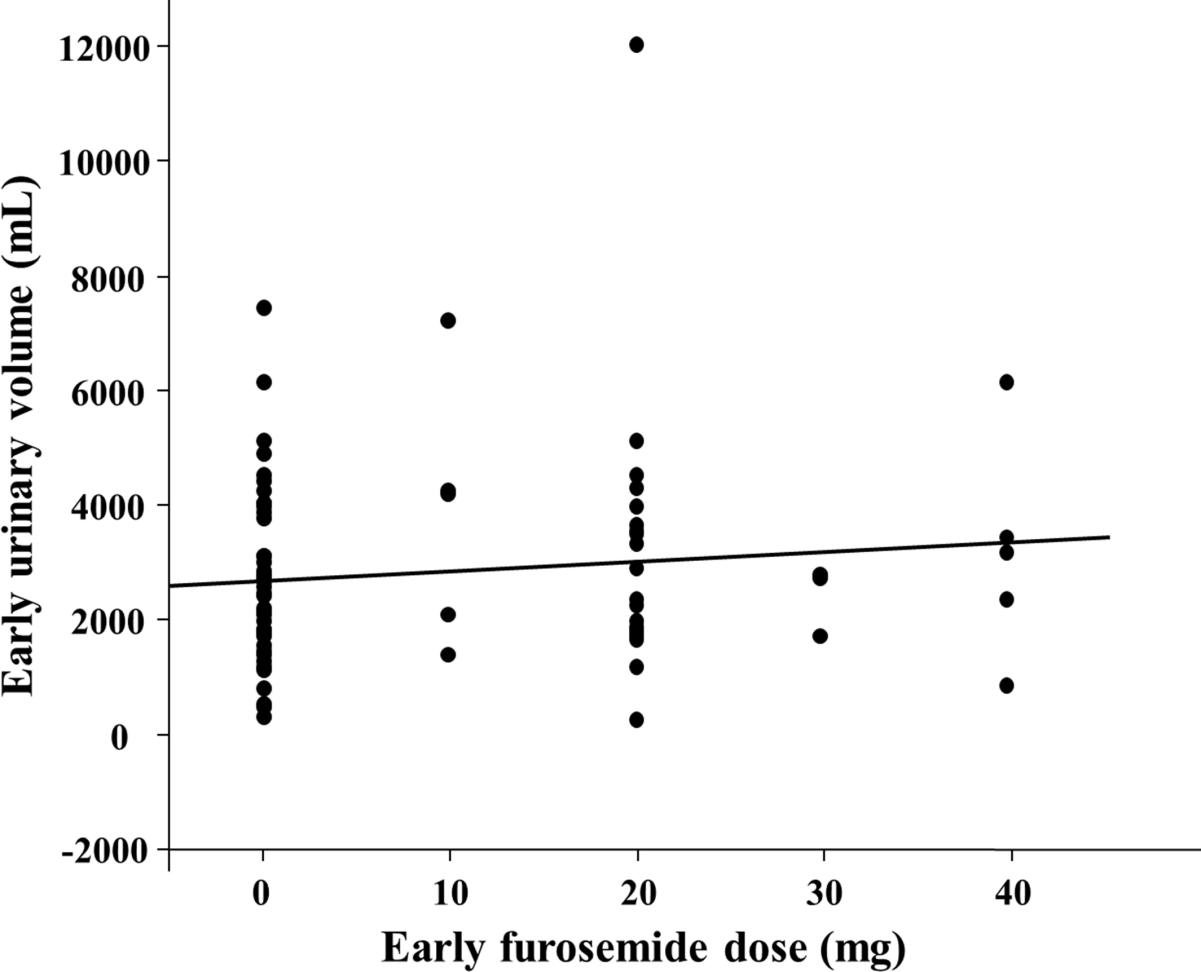
Relationship between early furosemide dose and early urinary volume.

Table 2 shows the in-hospital clinical outcomes and medications. The urinary volume during the early period was greater in the GR group than in the PR group. The lengths of the early period were similar between the two groups. The incidence of in-hospital death, cerebral infarction, hypotension, and RRT did not differ between the two groups. The incidence of WRF was significantly higher in the PR group than in the GR group. The administered carperitide dose on day 1 and day 2 did not significantly differ between the two groups. The rates of intravenous furosemide during hospitalization and the early intravenous furosemide doses were similar between the two groups. As shown in Fig 2, there was no correlation between early intravenous furosemide dose and urinary volume, with a Spearman’s rank correlation coefficient of 0.111 (*p* = 0.312).

**Table 2:** In-hospital clinical outcomes and medications.

	GR group	PR group	*p* value
	(n = 42)	(n = 43)	
In-hospital clinical outcomes			
Urinary volume for the early period, median (IQR), mL	3805 (2983–4520)	1831 (1338–2056)	<0.001
Length of the early period, median (IQR), h	34 (32–38)	34 (30–36)	0.355
Urinary volume / time, median (IQR), mL/h	63.7 (54.8–77.2)	29.7 (25.1–34.7)	<0.001
In-hospital death, n (%)	1 (2.4)	3 (7.0)	0.616
Cerebral infarction, n (%)	3 (7.1)	1 (2.3)	0.360
Hypotension, n (%)	3 (7.1)	5 (11.6)	0.713
RRT, n (%)	1 (2.4)	3 (7.0)	0.616
WRF, n (%)	9 (21.4)	19 (44.2)	0.026
NIPPV, n (%)	4 (9.5)	12 (27.9)	0.050
Medications during hospitalization			
Carperitide dose on day 1, median (IQR), μg/kg/min	0.025 (0.025–0.025)	0.025 (0.025–0.025)	0.480
Carperitide dose on day 2, median (IQR), μg /kg/min	0.025 (0.025–0.025)	0.025 (0.025–0.025)	1.000
Nitrate infusion, n (%)	10 (23.8)	10 (23.3)	0.952
ISDN, n (%)	3 (7.1)	2 (4.7)	0.676
NTG, n (%)	7 (16.7)	8 (18.6)	0.815
Catecholamine, n (%)	0 (0)	4 (9.3)	0.116
Intravenous furosemide for the early period, n (%)	20 (47.6)	15 (34.9)	0.233
Intravenous furosemide dose for the early period, median (IQR), mg	0 (0–20)	0 (0–20)	0.179
Furosemide dose in patients who received intravenous furosemide for the early period, median (IQR), mg	20 (20–30)	20 (20–20)	0.440
Intravenous furosemide during hospitalization, n (%)	24 (57.1)	22 (51.2)	0.580
Total intravenous furosemide dose, median (IQR), mg	15 (0–45)	20 (0–50)	0.598

GR, good diuretic responder; PR, poor diuretic responder; IQR, interquartile range; RRT, renal replacement therapy; WRF, worsening renal function; NIPPV, non-invasive positive pressure ventilation; ISDN, isosorbide dinitrate; NTG, nitroglycerin. The early period was defined as the period from admission until 24:00 of the next day of hospitalization.

### Predictors of diuretic response to carperitide

The results of a multivariate analysis incorporating significant risk factors from the univariate analyses showed that the statistically significant independent factors associated with poor diuretic response to carperitide were BMI (OR = 0.82, 95% CI 0.68–0.94, *p* = 0.004) and BUN (OR = 1.07, 95% CI 1.01–1.15, p = 0.018) on admission (Table 3).

**Table 3:** Univariate and multivariate analysis of factors related to PR.

	Univariate analysis			Multivariate analysis		
	Odds ratio (95% CI)		*p* value	Odds ratio (95% CI)		*p* value
Age	1.06	(1.02–1.11)	0.001	1.02	(0.98–1.07)	0.364
Male	0.94	(0.40–2.25)	0.897			
NYHA IV	2.18	(0.88–5.59)	0.092	1.65	(0.54–5.13)	0.376
Diabetes mellitus	1.30	(0.54–3.14)	0.561			
Old myocardial infarction	1.90	(0.75–4.99)	0.179			
BMI	0.83	(0.72–0.93)	<0.001	0.82	(0.68–0.94)	0.004
SBP	0.99	(0.98–1.01)	0.291			
LVEF	1.03	(0.99–1.06)	0.074	1.02	(0.98–1.06)	0.243
BUN	1.07	(1.02–1.12)	0.002	1.07	(1.01–1.15)	0.018
eGFR	0.98	(0.97–1.00)	0.100	1.01	(0.98–1.03)	0.587
Log NT-proBNP	1.39	(0.53–3.86)	0.507			
Oral furosemide dose on admission	1.01	(0.99–1.03)	0.517			
Intravenous furosemide dose for the early period	0.97	(0.94–1.01)	0.133			

PR, poor diuretic responder; CI, confidence interval; NYHA, New York Heart Association; BMI, body mass index; SBP, systolic blood pressure; LVEF, left ventricular ejection fraction; BUN, blood urea nitrogen; eGFR, estimated glomerular filtration rate; NT-proBNP, N-terminal pro-B-type natriuretic peptide.

### Diuretic response and outcome with carperitide

**Fig 3:**
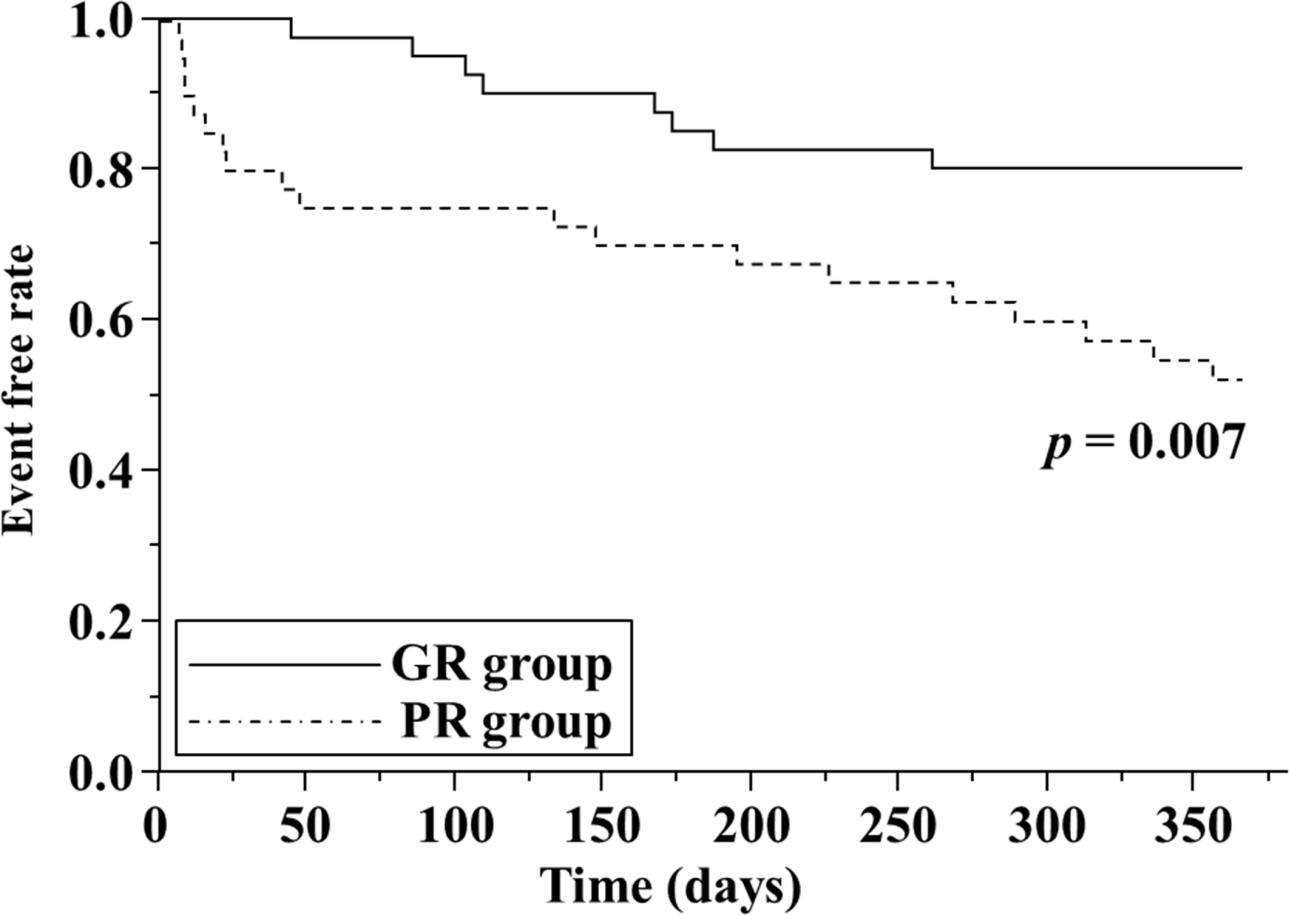
Kaplan–Meier curves for the composite outcome of all-cause mortality and re-hospitalization for worsening heart failure. GR, good diuretic responder; PR, poor diuretic responder.

Based on a Kaplan–Meier analysis, the composite outcome of all-cause mortality and re-hospitalization for worsening HF at 1 year was 20% (8/40) in the GR group and 48% (19/40) in the PR group. Log-rank test showed that PR group had a significantly poorer composite outcome compared with GR group (log-rank, *p* = 0.007; Fig 3).

## Discussion

To the best of our knowledge, this study is the first to investigate the diuretic response to carperitide in patients hospitalized for ADHF. Our study revealed that lower BMI and higher BUN level on admission were significant determinants of early poor diuretic response to carperitide, which was independent of early intravenous furosemide dose. In addition, early poor diuretic response to carperitide was associated with developing WRF and future all-cause mortality and re-hospitalization for worsening HF.

Diuretic response is a strong predictor of prognosis in patients hospitalized for ADHF. Testani et al. reported that poor diuretic response predicted poor prognosis above and beyond traditional prognostic factors in patients hospitalized for ADHF in a post-hoc analysis [3]. Especially, the assessment of early diuretic response that is a strong predictor of later diuretic response is important. It has been reported that poor diuretic response can be predicted by measuring early diuretic response [14]. However, most clinical trials on diuretic response have investigated response to furosemide which acts on the thick ascending loop of Henle to inhibit the Na_2_CL/K cotransporter. On the other hand, Carperitide, an atrial natriuretic peptide, inhibits the amiloride-sensitive sodium channels in the medullary collecting duct and decreases plasma renin levels, resulting in diuresis and natriuresis [15, 16]. Although small, open label trails have reported that carperitide improved hemodynamics and long-term prognosis in patients with ADHF [17, 18], several propensity score matched analyses have indicated that carperitide increased hospitalization costs and did not improve in-hospital mortality in patients hospitalized for acute HF [19–21]. More recently, a double-blind randomized controlled trial reported that carperitide did not improve short-term mortality despite significantly reducing the acute-phase pulmonary capillary wedge pressure compared with the placebo therapy [22]. Nonetheless, carperitide is recommended in the Japanese Circulation Society guidelines for acute HF treatment and is still frequently used as first-line treatment in Japan [9]. Therefore, we sought to investigate the effects of early diuretic response to carperitide in ADHF treatment.

The sub-analysis of ASCEND-HF suggested that poor diuretic response was predicted by lower BMI, lower BP, higher BUN, and lower received intravenous diuretic dose, which is almost similar to the predictors of poor response observed in the other studies [2]. In agreement with these reports, our study with carperitide showed that lower BMI and higher BUN level on admission were independent predictors of poor diuretic response. Low BMI may represent cardiac cachexia, which is related to worse prognosis and increased mortality. Patients with cardiac cachexia develop symptoms of HF with slight fluid retention, resulting in diuretic resistance. High BUN levels might reflect renal dysfunction and neurohumoral activation. BUN competitively binds the organic anion transporters, reducing diuretic availability in the tubule [1, 23].

Poor diuretic responder to carperitide had a higher WRF incidence in the present study. Traditionally, WRF occurs in 30%–50% of patients hospitalized for acute HF and is associated with increased mortality, prolonged length of stay, and increased risk of readmission [24]. Aronson et al. reported that early net fluid loss was a stronger indicator of WRF than was right atrial pressure in a sub-analysis of the VMAC study that investigated the effects of intravenous vasodilator therapy, including nesiritide [11]. The report showed that favorable net fluid loss during the first day after admission was associated with less incidence of WRF. Our results with carperitide were in agreement with those of the sub-analysis in the VMAC study. Our results, thus, suggest that early diuretic response should be considered as the predictor of WRF in patients admitted for ADHF.

We showed that furosemide dose was not associated with urinary volume and was independent of the predictors of poor diuretic response. In addition, there was no difference in the total intravenous furosemide dose during hospitalization between the two groups (median dose, 15 mg vs. 20 mg). The furosemide dose was very small compared with the previous report [2]. Carperitide may have contributed to a reduction in the intravenous furosemide dose. It has been reported that high dose of furosemide is associated with poor prognosis. However, more recently, high-dose furosemide was found to not always be associated with worse outcomes in patients hospitalized for acute HF without poor diuretic response and persistent signs of congestion [25]. We demonstrated that poor diuretic response was associated with poor future prognosis in patients using carperitide who received a low introduction rate and low-dose intravenous furosemide in treatment of ADHF. These results suggest that the prognosis of HF might be determined by diuretic response independent of furosemide dose and the site of diuretic action.

### Limitations

The results of the current study should be interpreted in the context of the following limitations. First, this was a single-center retrospective study and follow-up was limited to the in-hospital treatment. The most common problem with analysis of retrospective observational data is that inadequate validity of comparisons leads to incorrect interpretation. We cannot exclude the possibility that residual measured and/or unmeasured confounders may have influenced our observations. Second, we excluded patients lacking urinary catheter because urinary volume measurements are not reliable without a urinary catheter. We speculated that the reasons why attending physicians did not provide urinary catheters or continue their use were that the patients did not present with severe symptoms, rejected the provided urinary catheter, and had organic and functional pathologies that made it difficult to place a urinary catheter (e.g., urethral stricture, urogenital infection, delirium, dementia). Thus, we cannot completely exclude the possibility of selection bias. Third, we did not include information on fluid intake because of missing values. Finally, we did not define WRF as the absolute change in creatinine level. WRF is typically defined as an increase in serum creatinine of >0.3 mg/dL from the baseline value. However, this classic definition of creatinine could cause either an underestimation of WRF in patients with low serum creatinine or an overestimation in those with high serum creatinine. In addition, serum creatinine is strongly influenced by age and muscle mass. Therefore, we chose a definition of a decrease in eGFR during hospitalization of >25%, which has been used by other investigators [11–13]. Further prospective studies are needed to clarify the effects of diuretic response to carperitide.

## Conclusions

BMI and BUN levels on admission were significant determinants of early poor diuretic response to carperitide. Poor diuretic response to carperitide resulted in developing WRF and increased all-cause mortality and re-hospitalization for worsening HF.

## References

[1] ter Maaten JM, Valente MA, Damman K, Hillege HL, Navis G, Voors AA. Diuretic response in acute heart failure-pathophysiology, evaluation, and therapy. Nature reviews Cardiology. 2015;12(3):184–92. Epub 2015/01/07. doi: 10.1038/nrcardio.2014.215 . 25560378 10.1038/nrcardio.2014.215

[2] Gottlieb SS, Stebbins A, Voors AA, Hasselblad V, Ezekowitz JA, Califf RM, et al Effects of nesiritide and predictors of urine output in acute decompensated heart failure: results from ASCEND-HF (acute study of clinical effectiveness of nesiritide and decompensated heart failure). Journal of the American College of Cardiology. 2013;62(13):1177–83. Epub 2013/06/12. doi: 10.1016/j.jacc.2013.04.073 . 23747790 10.1016/j.jacc.2013.04.073

[3] Testani JM, Brisco MA, Turner JM, Spatz ES, Bellumkonda L, Parikh CR, et al Loop diuretic efficiency: a metric of diuretic responsiveness with prognostic importance in acute decompensated heart failure. Circulation Heart failure. 2014;7(2):261–70. Epub 2014/01/01. doi: 10.1161/CIRCHEARTFAILURE.113.000895 ; PubMed Central PMCID: PMCPmc4386906. 24379278 10.1161/CIRCHEARTFAILURE.113.000895 PMC4386906

[4] Palazzuoli A, Testani JM, Ruocco G, Pellegrini M, Ronco C, Nuti R. Different diuretic dose and response in acute decompensated heart failure: Clinical characteristics and prognostic significance. International journal of cardiology. 2016;224:213–9. doi: 10.1016/j.ijcard.2016.09.005 27657476 10.1016/j.ijcard.2016.09.005

[5] ter Maaten JM, Dunning AM, Valente MA, Damman K, Ezekowitz JA, Califf RM, et al Diuretic response in acute heart failure-an analysis from ASCEND-HF. American heart journal. 2015;170(2):313–21. Epub 2015/08/25. doi: 10.1016/j.ahj.2015.05.003 . 26299229 10.1016/j.ahj.2015.05.003

[6] Group JCSJW. Guidelines for Treatment of Acute Heart Failure (JCS 2011). Circulation Journal. 2013;77(8):2157–201. doi: 10.1253/circj.CJ-66-0068 23759659 10.1253/circj.cj-66-0068

[7] Okuhara Y, Hirotani S, Ando T, Nishimura K, Orihara Y, Komamura K, et al Comparison of salt with low-dose furosemide and carperitide for treating acute decompensated heart failure: a single-center retrospective cohort study. Heart and vessels. 2017;32(4):419–27. Epub 2016/07/30. doi: 10.1007/s00380-016-0883-1 . 27469321 10.1007/s00380-016-0883-1

[8] Nakano Y, Mizuno T, Niwa T, Mukai K, Wakabayashi H, Watanabe A, et al Impact of Continuous Administration of Tolvaptan on Preventing Medium-Term Worsening Renal Function and Long-Term Adverse Events in Heart Failure Patients with Chronic Kidney Disease. International Heart Journal. 2018;advpub. doi: 10.1536/ihj.16-625 29332911 10.1536/ihj.16-625

[9] Sato N, Kajimoto K, Asai K, Mizuno M, Minami Y, Nagashima M, et al Acute decompensated heart failure syndromes (ATTEND) registry. A prospective observational multicenter cohort study: rationale, design, and preliminary data. American heart journal. 2010;159(6):949–55 e1. doi: 10.1016/j.ahj.2010.03.019 . 20569705 10.1016/j.ahj.2010.03.019

[10] Matsuo S, Imai E, Horio M, Yasuda Y, Tomita K, Nitta K, et al Revised equations for estimated GFR from serum creatinine in Japan. American journal of kidney diseases: the official journal of the National Kidney Foundation. 2009;53(6):982–92. Epub 2009/04/03. doi: 10.1053/j.ajkd.2008.12.034 . 19339088 10.1053/j.ajkd.2008.12.034

[11] Aronson D, Abassi Z, Allon E, Burger AJ. Fluid loss, venous congestion, and worsening renal function in acute decompensated heart failure. European journal of heart failure. 2013;15(6):637–43. Epub 2013/03/12. doi: 10.1093/eurjhf/hft036 . 23475780 10.1093/eurjhf/hft036

[12] Lazaros G, Tsiachris D, Tousoulis D, Patialiakas A, Dimitriadis K, Roussos D, et al In-hospital worsening renal function is an independent predictor of one-year mortality in patients with acute myocardial infarction. International journal of cardiology. 2012;155(1):97–101. doi: 10.1016/j.ijcard.2010.10.024 21078526 10.1016/j.ijcard.2010.10.024

[13] O'Connor CM, Starling RC, Hernandez AF, Armstrong PW, Dickstein K, Hasselblad V, et al Effect of nesiritide in patients with acute decompensated heart failure. The New England journal of medicine. 2011;365(1):32–43. Epub 2011/07/08. doi: 10.1056/NEJMoa1100171 . 21732835 10.1056/NEJMoa1100171

[14] ter Maaten JM, Valente MA, Metra M, Bruno N, O'Connor CM, Ponikowski P, et al A combined clinical and biomarker approach to predict diuretic response in acute heart failure. Clinical research in cardiology: official journal of the German Cardiac Society. 2016;105(2):145–53. Epub 2015/08/19. doi: 10.1007/s00392-015-0896-2 ; PubMed Central PMCID: PMCPmc4735256. 26280875 10.1007/s00392-015-0896-2 PMC4735256

[15] Schreiner GF, Protter AA. B-type natriuretic peptide for the treatment of congestive heart failure. Current Opinion in Pharmacology. 2002;2(2):142–7. http://dx.doi.org/10.1016/S1471-4892(02)00142-X. 11950624 10.1016/s1471-4892(02)00142-x

[16] Ishikawa C, Tsutamoto T, Wada A, Fujii M, Ohno K, Sakai H, et al Inhibition of aldosterone and endothelin-1 by carperitide was attenuated with more than 1 week of infusion in patients with congestive heart failure. Journal of cardiovascular pharmacology. 2005;46(4):513–8. Epub 2005/09/15. . 16160606 10.1097/01.fjc.0000177980.83810.2e

[17] Kitashiro S, Sugiura T, Takayama Y, Tsuka Y, Izuoka T, Tokunaga S, et al Long-term administration of atrial natriuretic peptide in patients with acute heart failure. Journal of cardiovascular pharmacology. 1999;33(6):948–52. Epub 1999/06/15. . 10367599 10.1097/00005344-199906000-00016

[18] Hata N, Seino Y, Tsutamoto T, Hiramitsu S, Kaneko N, Yoshikawa T, et al Effects of carperitide on the long-term prognosis of patients with acute decompensated chronic heart failure: the PROTECT multicenter randomized controlled study. Circulation journal: official journal of the Japanese Circulation Society. 2008;72(11):1787–93. Epub 2008/09/25. . 18812677 10.1253/circj.cj-08-0130

[19] Mizuno A, Iguchi H, Sawada Y, Hurley M, Nomura H, Hayashi K, et al The impact of carperitide usage on the cost of hospitalization and outcome in patients with acute heart failure: High value care vs. low value care campaign in Japan. International journal of cardiology. 2017;241:243–8. Epub 2017/05/10. doi: 10.1016/j.ijcard.2017.04.078 . 28476514 10.1016/j.ijcard.2017.04.078

[20] Ogiso M, Isogai T, Okabe Y, Ito K, Tsuji M, Tanaka H. Effect of carperitide on in-hospital mortality of patients admitted for heart failure: propensity score analyses. Heart and vessels. 2017;32(8):916–25. Epub 2017/02/22. doi: 10.1007/s00380-017-0952-0 . 28220240 10.1007/s00380-017-0952-0

[21] Matsue Y, Kagiyama N, Yoshida K, Kume T, Okura H, Suzuki M, et al Carperitide Is Associated With Increased In-Hospital Mortality in Acute Heart Failure: A Propensity Score-Matched Analysis. Journal of cardiac failure. 2015;21(11):859–64. Epub 2015/05/23. doi: 10.1016/j.cardfail.2015.05.007 . 25999241 10.1016/j.cardfail.2015.05.007

[22] Wang G, Wang P, Li Y, Liu W, Bai S, Zhen Y, et al Efficacy and Safety of 1-Hour Infusion of Recombinant Human Atrial Natriuretic Peptide in Patients With Acute Decompensated Heart Failure: A Phase III, Randomized, Double-Blind, Placebo-Controlled, Multicenter Trial. Medicine. 2016;95(9):e2947 doi: 10.1097/MD.0000000000002947 PubMed PMID: PMC4782891. 26945407 10.1097/MD.0000000000002947 PMC4782891

[23] Rubio-Gracia J, Demissei BG, ter Maaten JM, Cleland JG, O'Connor CM, Metra M, et al Prevalence, predictors and clinical outcome of residual congestion in acute decompensated heart failure. International journal of cardiology. 258:185–91. doi: 10.1016/j.ijcard.2018.01.067 29544928 10.1016/j.ijcard.2018.01.067

[24] Schmieder RE, Mitrovic V, Hengstenberg C. Renal impairment and worsening of renal function in acute heart failure: can new therapies help? The potential role of serelaxin. Clinical research in cardiology: official journal of the German Cardiac Society. 2015;104(8):621–31. Epub 2015/03/20. doi: 10.1007/s00392-015-0839-y . 25787721 10.1007/s00392-015-0839-y

[25] Hanberg JS, Tang WHW, Wilson FP, Coca SG, Ahmad T, Brisco MA, et al An exploratory analysis of the competing effects of aggressive decongestion and high-dose loop diuretic therapy in the DOSE trial. International journal of cardiology. 2017;241(Supplement C):277–82. https://doi.org/10.1016/j.ijcard.2017.03.114. 28392080 10.1016/j.ijcard.2017.03.114 PMC5471358

